# Generation of Nonhuman Primate Model of Cone Dysfunction through *In Situ* AAV-Mediated *CNGB3* Ablation

**DOI:** 10.1016/j.omtm.2020.08.007

**Published:** 2020-08-08

**Authors:** Qiang Lin, Ji-Neng Lv, Kun-Chao Wu, Chang-Jun Zhang, Qin Liu, Zi-Bing Jin

**Affiliations:** 1 Laboratory of Stem Cell & Retinal Regeneration, Division of Ophthalmic Genetics, The Eye Hospital, Wenzhou Medical University, Wenzhou 325027, China; 2 Ocular Genomics Institute, Massachusetts Eye and Ear Infirmary, Boston, MA, USA; 3 Department of Ophthalmology, Harvard Medical School, Boston, MA, USA; 4 Beijing Institute of Ophthalmology, Beijing Tongren Eye Center, Beijing Tongren Hospital, Capital Medical University, Beijing Ophthalmology & Visual Science Key Laboratory, Beijing 100730, China; 5 Beijing Advanced Innovation Center for Big Data-Based Precision Medicine, Beihang University & Capital Medical University, Beijing Tongren Hospital, Beijing 100730, China

## Abstract

A major challenge to the development of therapies for human retinal degenerative diseases is the lack of an ideal preclinical model because of the physiological differences between humans and most model animals. Despite the successful generation of a primate model through germline knockout of a disease-causing gene, the major issues restricting modeling in nonhuman primates (NHPs) are their relatively long lifespan, lengthy gestation, and dominant pattern of singleton births. Herein, we generated three cynomolgus macaques with macular *in situ* knockout by subretinal delivery of an adeno-associated virus (AAV)-mediated CRISPR-Cas9 system targeting *CNGB3*, the gene responsible for achromatopsia. The *in vivo* targeting efficiency of CRISPR-Cas9 was 12%–14%, as shown by both immunohistochemistry and single-cell transcriptomic analysis. Through clinical ophthalmic examinations, we observed a reduced response of electroretinogram in the central retina, which corresponds to a somatic disruption of CNGB3. In addition, we did not detect CRISPR-Cas9 residue in the heart, liver, spleen, kidney, brain, testis, or blood a year after administration. In conclusion, we successfully generated a NHP model of cone photoreceptor dysfunction in the central retina using an *in situ* CNGB3-knockout strategy.

## Graphical Abstract

**Figure:**
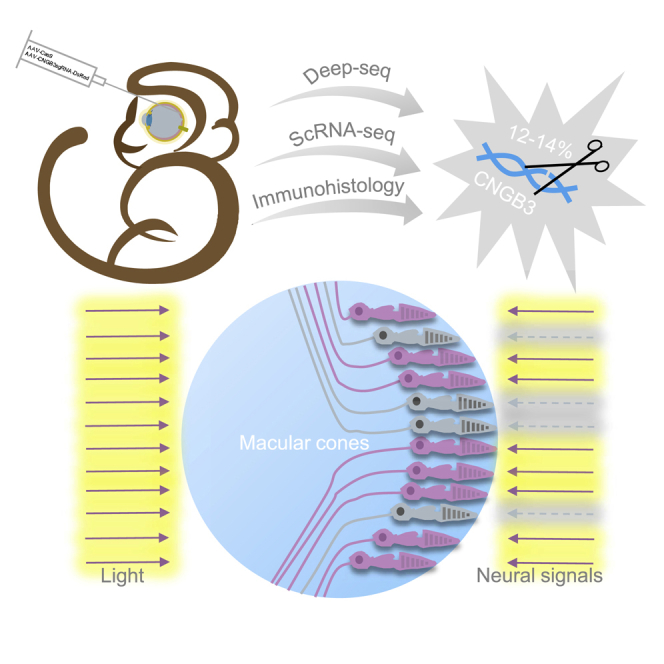

## Teaser

Jin and colleagues delivered AAV-mediated CRISPR-cas9 targeting a cone-specific gene, *CNGB3*, to rapidly generate a non-human primate model with achromatopsia. This study provides a new approach and insights to establish a non-human primate model recapitulating human macular degeneration.

## Introduction

Vision with high spatial resolution of color originates in the macula, a cone-photoreceptor-enriched region in the retina. Macula-related degeneration, comprising a wide spectrum of retinal diseases, is the leading cause of inevitable blindness in humans worldwide. Achromatopsia, a congenital recessive disorder, is caused by genetic mutations that trigger cone photoreceptor dysfunction.^1^, ^2^, ^3^, ^4^ The disease is characterized by a lack of color discrimination, low visual acuity, photophobia, and nystagmus.^5^ So far, several disease-causing genes have been described in patients with achromatopsia.^6^, ^7^, ^8^, ^9^, ^10^, ^11^, ^12^, ^13^, ^14^ Mutations in patients with achromatopsia and defective cone cyclic-nucleotide-gated (CNG) channel subunits are particularly prevalent^15^ because mutations in *CNGB3* account for 45.2% patients.^16^

In mammals, only primates have a macula that is closely related to that of humans, with a concaviclivate fovea, which contains the highest density of cone photoreceptors.^17^ Non-human primates (NHPs) offer the unique advantages of phylogenetic proximity, conserved gene maps, and similarity of physiology and disease susceptibility with humans, making NHPs particularly suitable for genetic research on complex physiological and behavioral phenotypes relevant to human diseases.^18^ However, the major issues restricting disease modeling in NHPs are long lifespan, lengthy gestational period, and generally giving birth to singleton offspring.^18^

The CRISPR-Cas9 system has emerged as a revolutionary tool for generating loss-of-function mutations.^19^, ^20^, ^21^, ^22^ In the present study, we generated a *CNGB3* somatic knockout model in NHPs to recapitulate achromatopsia through subretinal injection of adeno-associated virus 9 (AAV9) with CRISPR-Cas9 targeting *CNGB3*. This new model exhibited a reduced amplitude in the central retina, as shown by multifocal electroretinogram (mfERG). We further investigated the resultant gene-editing efficiency in photoreceptors and transcriptomic changes. Our results provide a new approach to generate a disease model in NHPs and new insight into therapeutic schemes for genetic maculopathy.

## Results

### CRISPR-Cas9 Cleaved the *CNGB3* Locus in COS-7 Cells

*CNGB3* has two mRNA isoforms (Figure S1A). The exons were rearranged after the sixth exon in these transcripts, and exon 6 is the first of the first-transmembrane domain of CNGB3. Exon 6 of *CNGB3* was selected as the target, thus ablating all *CNGB3* isoforms. To investigate the efficiency of CRISPR-Cas9-mediated cleavage at the *CNGB3* locus, we tested three single-guide RNAs (sgRNAs; CNGB3sg1, CNGB3sg2, and CNGB3sg3) *in vitro* (Figure S1A). Targeting efficiencies of 5.2%, 9.6%, and 28% were observed for CNGB3sg1, CNGB3sg2, and CNGB3sg3, respectively, as determined by T7EN1 assay and Sanger sequencing (Figures S1B and S1C). Thereby, CNGB3sg3 was chosen for the *in vivo* study.

### Subretinal Injection of AAV9-SpCas9/*CNGB3*-sgRNA Components in Cynomolgus Monkeys

**Figure 1:**
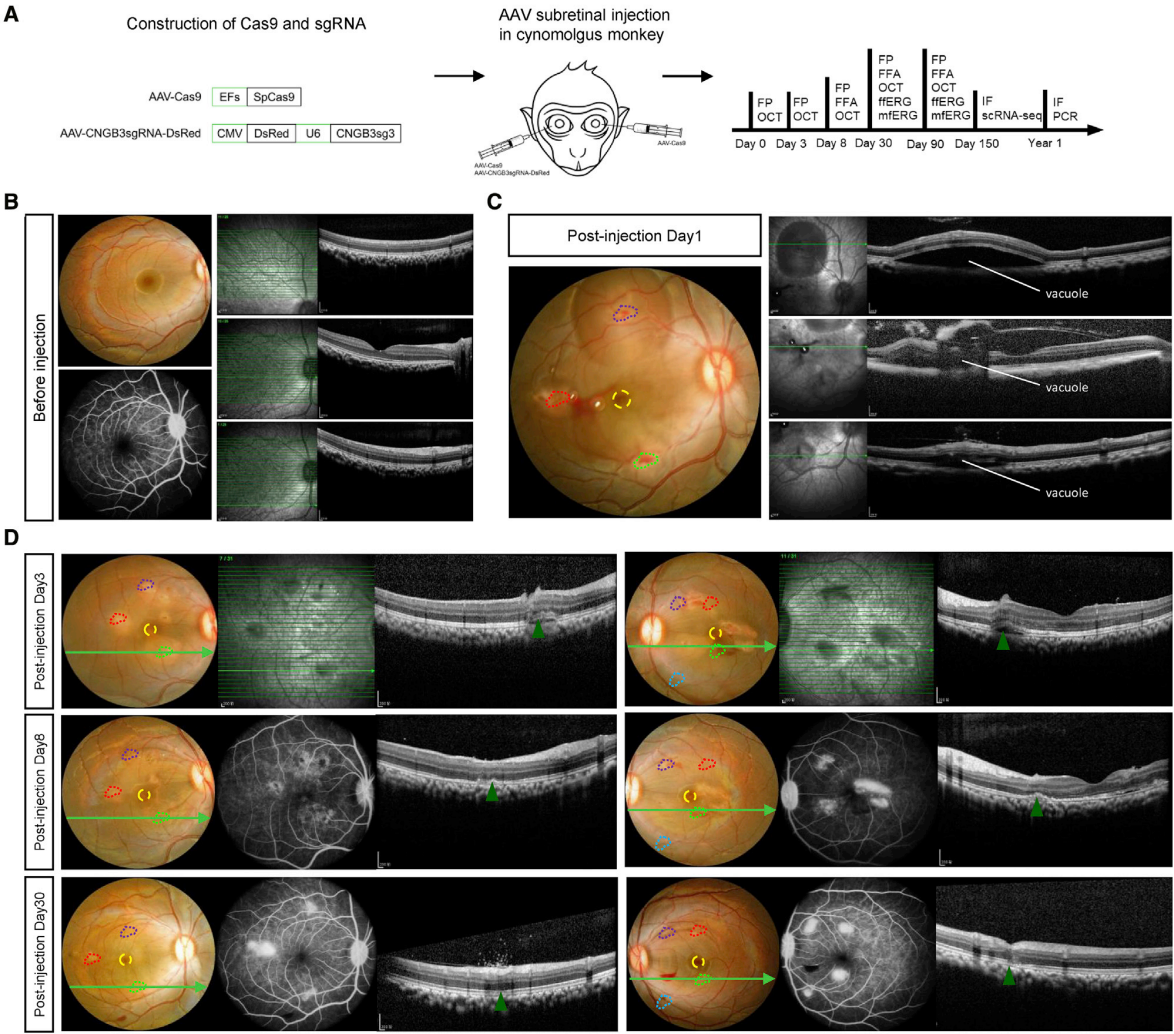
Scheme of Subretinal AAV Injection (A) Scheme of AAV9-based subretinal administration. (B) Representative FP, FFA, and OCT images of the right eye, showing the structure of the retina before AAV injection. (C) Representative FP and OCT images monitor the puncture sites and AAV vacuoles on injection day 1. Punctures are indicated by dotted colored polygons, and the foveae are indicated with dotted circles in yellow. (D) Representative FP, FFA, and OCT images indicate the recovery of the retina after puncture at days 3, 8, and 30. Green arrows in the FP images show the imaging site of the OCT, and green triangles in the OCT images indicate the puncture sites.

To knock out the *CNGB3* gene in the macular region of monkeys *in vivo*, we delivered *Streptococcus pyogenes* Cas9 (SpCas9) and CNGB3sg3 with two separate adeno-associated virus 9 (AAV9) vectors (Figure 1A), and 100 μL of premixed AAVs that contained 3E−10 viral genomes (vg) of AAV9-SpCas9 and 3E−10 vg of AAV9-CNGB3-sgRNA3-DsRed was injected subretinally into three separate sites (paramacular area, upper vascular arch, and the inferior vascular arch) of the right eyes in three cynomolgus monkeys (age, 4 year). The left eyes were injected with 100 μL AAV9-SpCas9 at the corresponding sites as controls. Fundus photography (FP) and optical coherence tomography (OCT) images were recorded before injection and immediately after injection and at post-injection days 3, 8, and 30 to monitor the status of injection (Figures 1B–1D and S2). Immediately after injection, vacuoles surrounding the puncture points suggested successful delivery of AAVs into the subretinal space (Figures 1C and S2B). The vacuoles gradually diminished, and the retina reattached on post-injection day 8 (Figure 1D). All three monkeys underwent successful delivery of AAVs into the subretinal space of both right and left eyes.

### *In Situ* CNGB3 Knockout in Cone Photoreceptors of Central Retina

**Figure 2:**
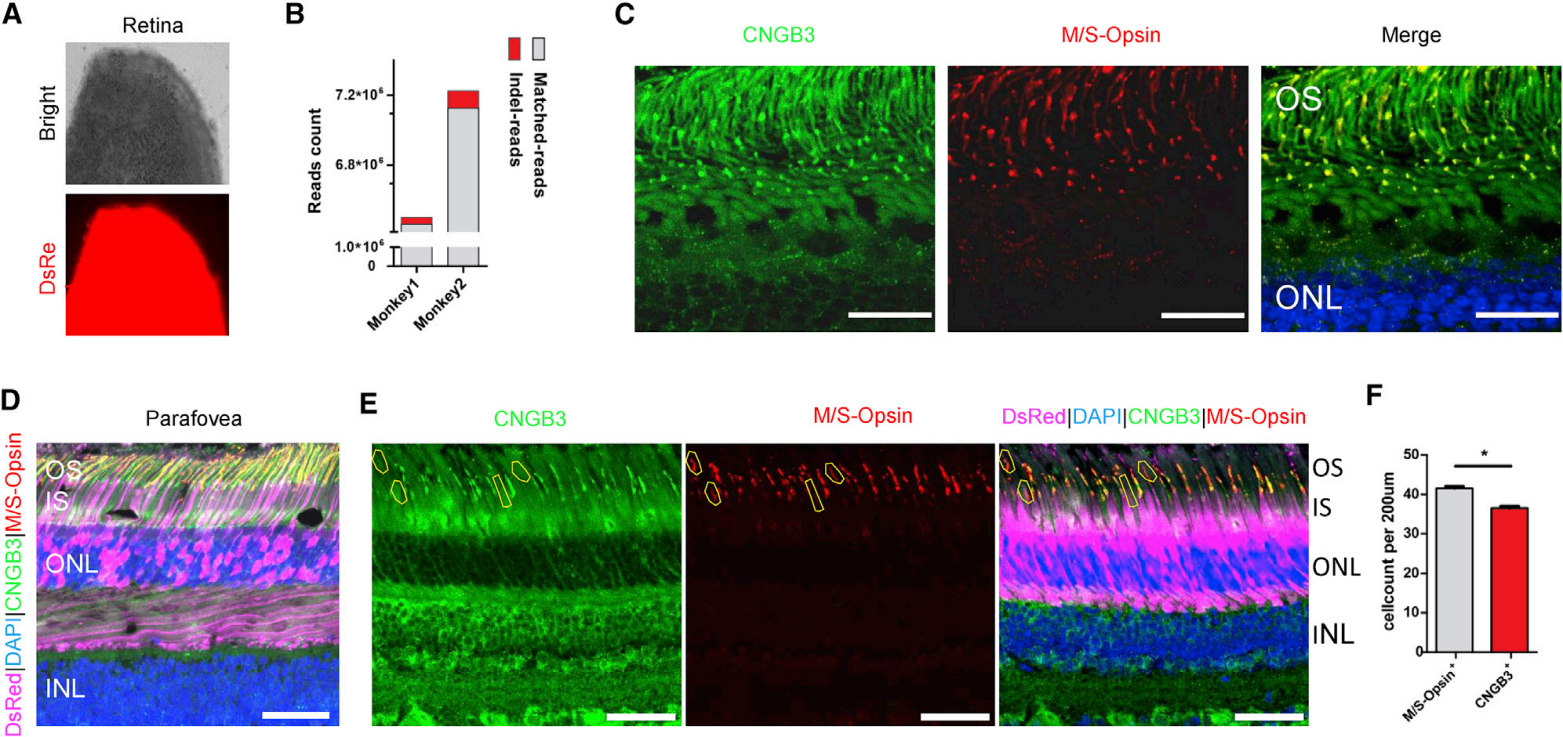
*CNGB3* Targeting Efficiency Tests in Monkey Cone Photoreceptors Infected with AAVs (A) Dissected retina showing strong DsRed signal, revealing the expression of the CRISPR-Cas9 system. (B) Statistics of indel reads and matched reads in deep sequencing show the targeting efficiency at the genome level. (C) Coimmunostaining of CNGB3 and M/S-opsin in the control retina. These proteins localized in the outer segments of the photoreceptors and showed strong overlapping signals. OS, outer segment; ONL, outer nuclear layer. The scale bar representing 25 μm is indicated at the bottom right of the figure. (D) Representative staining image of cone-specific photoreceptor antibodies, M/S-opsin and CNGB3, in the macular region, which shows the specificity of AAV9. AAV-*CNGB3*sgRNA-DsRed is indicated in pink. OS, outer segment; IS inner segment; ONL, outer nuclear layer; INL, inner nuclear layer; GCL, ganglion cell layer. The scale bar representing 50 μm is indicated at the bottom right of the figure. (E) Representative image showing *CNGB3* knockout cones, as indicated by immunofluorescence. CNGB3 (green) and M/S-opsin (red) were used to survey the knockout efficiency in cones. Those cones are marked with yellow polygons. The scale bar representing 50 μm is indicated bottom right in the figure. (F) Statistics for (E). Each column represents the means of n = 2 animals. The p value was calculated with t tests (t = 7.071, df = 2) and is indicated in the figure. ∗p < 0.05, ∗∗p < 0.01, ∗∗∗p < 0.001; n.s, no significance.

To evaluate the targeting efficiency of AAV9-SpCas9-CNGB3 sgRNA3, transduced and control retinas were obtained and analyzed. Dissected retina that was positive for DsRed was confirmed, indicating expression from the AAV9-sgRNA vector, which could guide the dissection (Figure 2A). The DsRed^+^ retinal region was dissected into three parts: the central retina containing the fovea (Figure S3, shown as yellow ellipse) was used to perform the immunohistochemistry, and the other two parts (Figure S3, shown as red circle) were used in the isolation of single cells to perform deep sequencing. Paramacular retina (labeled with a red circle) was preferentially used to isolate single cells, and the remaining tissue was used for targeted deep sequencing. High-throughput sequencing showed 0.5% and 1.3% indel reads for monkeys 1 and 2, respectively (Figure 2B).

To evaluate somatic CNGB3 depletion in the macular area, we tested the specificity of two cone markers, CNGB3 and M/S-Opsin, in the central retina of the left eye. As expected, CNGB3 colocalized well with M/S-opsin in the outer segment of cone photoreceptors (Figure 2C). This colocalization could be used to subsequently calculate the targeting efficiency. We found that DsRed was expressed exclusively (indicated in pink) in the photoreceptor and retinal pigment epithelium (RPE) layer of the injection area, implying successful delivery of CRISPR-Cas9 components to the macula (Figure 2D). To estimate the proportion of cones with *CNGB3* knocked out, we calculated the number of M/S-opsin^+^/CNGB3^−^ cones in the sectioned retina (Figures 2E and 2F). As shown in the figures, four to six cells out of 40–42 DsRed^+^ cells had lower levels of *CNGB3* (200 μm). Because the AAVs infected cells randomly, we inferred that the targeting efficiency in infected cells was 12.2%, suggesting that subretinal delivery of AAV-mediated CRISPR-Cas9 and sgRNA successfully generated a partial knockout of CNGB3.

### Single-Cell Sequencing of Isolated Cones Validated Partial Ablation of the *CNGB3* Gene

**Figure 3:**
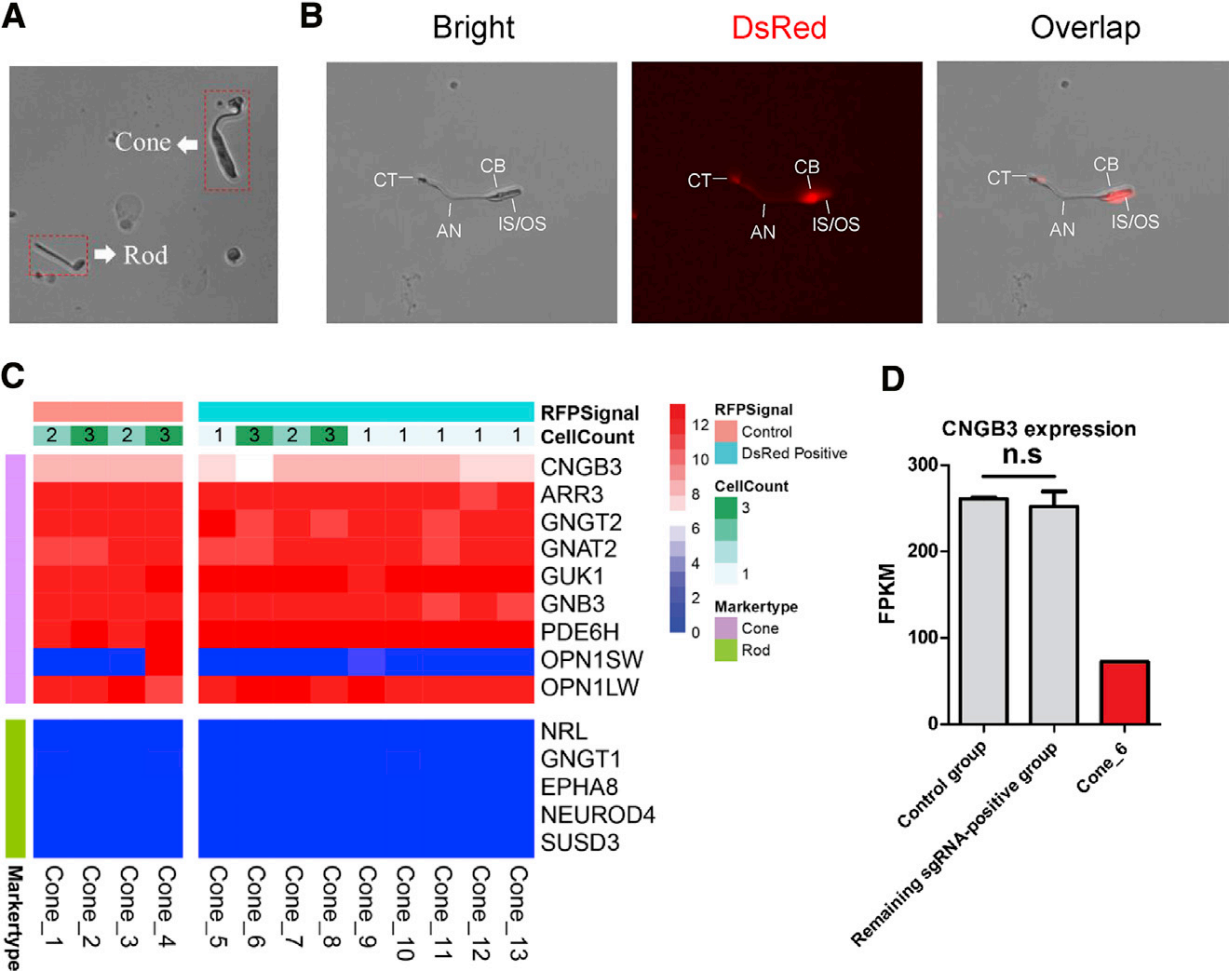
Single-Cell Sequencing (A) Representative image of the morphology of cones and rods in a single-cell suspension under EVOS. (B) Representative images of a single cone with intact structure and DsRed signal. IS/OS, inner segment/outer segment; CB, cell body; AN, axon; CT, cone terminal. (C) Heatmap of single-cone groups with typical cone and rod markers. The scalar presents log_2_ (fragments per kilobase of transcript per million mapped reads [FPKM] + 1). (D) *CNGB3* expression in single-cone groups. The p value was calculated with a t test (t = 0.124, df = 3) and is indicated in the figure. n.s, no significance.

To determine transcriptional changes of *CNGB3* in cone photoreceptors, single cells were isolated from the dissected retina for single-cell RNA sequencing (scRNA-seq) (Figure S3, shown in red).^23^, ^24^, ^25^ Based on the cell morphology in the single-cell suspension, cones were easily distinguished from rods and other retinal cells (Figure 3A). We manually picked 24 cones from the retina and divided them into 13 groups, termed cone_1 to cone_13, which were subjected to scRNA-seq by Smart-seq2 (Table S1). Each group included at least one and as many as three cones, and only those with intact cellular cone structure were selected (Figure 3B). Among them, those showing a DsRed signal were captured as sgRNA^+^ groups (cone_5 to cone_13; 14 cones in total), whereas the others were considered a control group (cone_1 to cone_4; 10 cones in total) (Table S1). scRNA-seq revealed a typical cone photoreceptor profile (Figure 3C). Then, we examined *CNGB3* expression in these groups. Notably, the *CNGB3* expression level in cone_6 was decreased to one-third of the level observed in the control groups, whereas there was no significant difference between the remaining sgRNA^+^ groups and the control groups (Figure 3D). Because loss-of-function of *CNGB3* causes achromatopsia and there was no disease phenotype in both *CNGB3*^+/+^ and *CNGB3*^+/−^ mice,^26^ we inferred that the decrease in *CNGB3* resulted from successful biallelic deletion of *CNGB3* in two of three cones in cone_6. Two of the 14 DsRed^+^ cones showed decreased levels of *CNGB3*, indicating that the targeting efficiency was 14.3%, which is consistent with the results from retinal immunohistochemistry. Then, we compared the profiling of the control groups and remaining sgRNA^+^ groups (cone_6 excluded) by edgeR software. No significant difference in gene expression was detected, suggesting that CRISPR-Cas9 expression did not make any difference (Figure S4A). We also searched the protein interaction database (STRING) and selected a set of 21 genes involved in the phototransduction that was predicted to interact with *CNGB3* in humans and macaques (Table S2). However, none of them showed significant differential expression in cone_6 (Figure S4B). Taken together, these results demonstrated partial knockout of *CNGB3* gene in cone photoreceptors, consistent with the findings from retinal immunohistochemistry.

### Retinal Phenotypes of CNGB3 Dysfunction in the NHP Model

**Figure 4:**
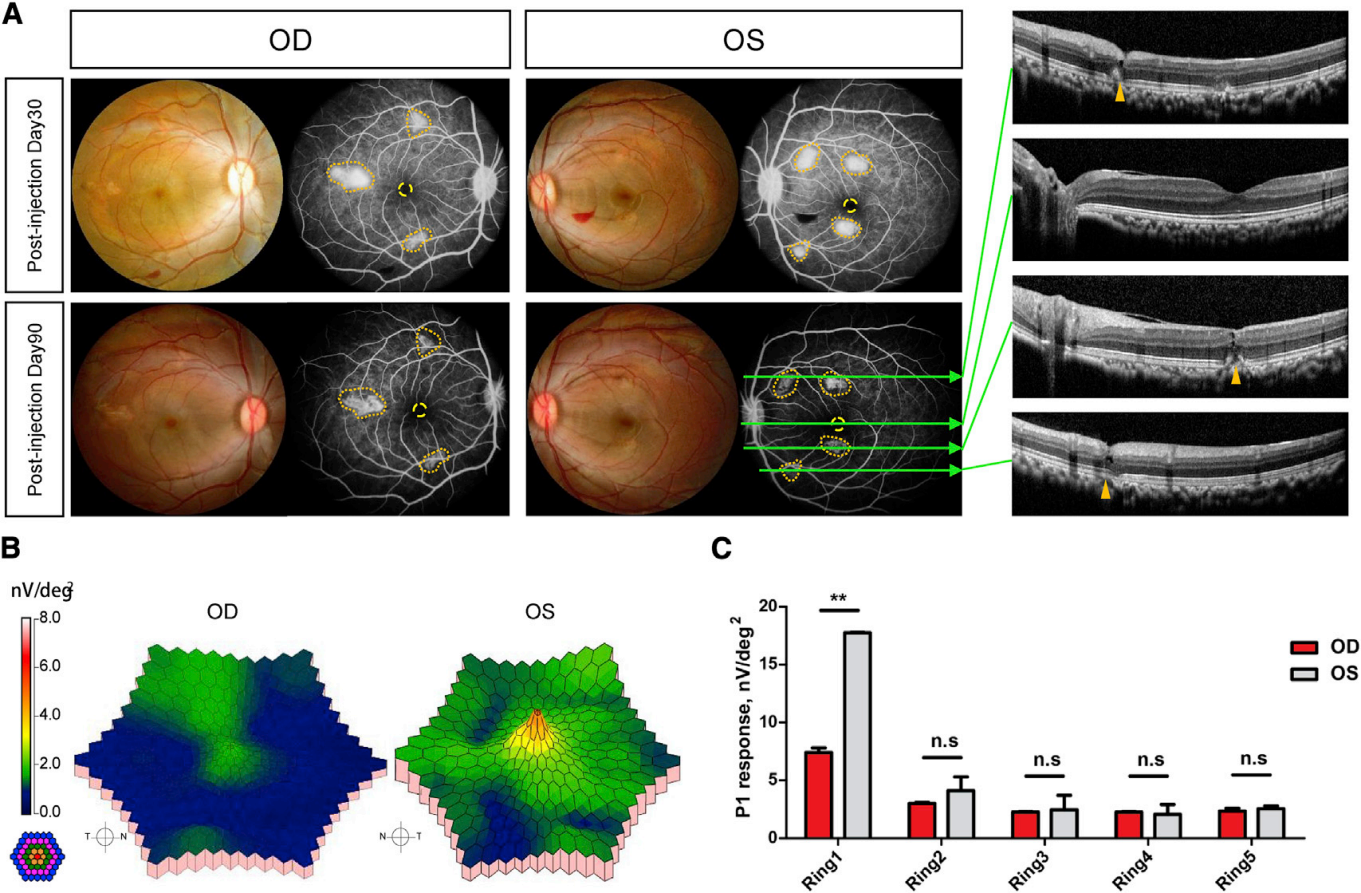
Ophthalmic Investigation of NHP Models (A) Representative FP and FFA images of monkey 2 at both postinjection days 30 and 90. Dotted polygons in brownish yellow indicate the leakage region of the FFA. Green arrows in the FFA images show imaging sites of the OCT. The OCT images reveal the puncture sites and the fovea of monkey 2 at postinjection day 90. (B) Representative 3D topography of monkey 2 mfERG root-mean-square (RMS) density. The colored rings on the bottom left indicate the stimulation range (61 hexagons) in mfERG. OD, right eye; OS, left eye. (C) Statistics of the P1 response in each ring. Each column represents the means of n = 2 animals. The p value was calculated with a t test (t = 0.9135, df = 2) and is indicated in the figure. ∗p < 0.05, ∗∗p < 0.01, ∗∗∗p < 0.001; n.s, no significance.

To validate the phenotypic consequence of the somatic partial knockout of *CNGB3* in NHP retinas, we performed clinical ophthalmic examinations including fundus photography, fundus fluorescein angiography (FFA), OCT, full-field electroretinogram (ffERG), and mfERG. FFA exhibited obvious leakage at the injection sites, and OCT confirmed the spot damage (Figure 4A). The results of the ffERG appeared normal (Figure S5), indicating the function of the overall retina was not significantly affected. Although mfERG showed reduced amplitude in the central retina at D90 (Figures 4B and 4C), implying the macular function was partially affected. To exclude the injecting damage on the response in mfERG, we assessed the position of the puncture site and the abnormal response regions in the mfERG of the left eye. We validated that the abnormal ERG response in foveal region was not caused by the physical damage (Figure 4A). In addition, we confirmed other puncture sites and abnormal response regions colocalized well, suggesting that the abnormal response was caused by structural damage from the puncture (Figures 4A and 4B, yellow polygon). We conclude that the reduction of mfERG response in the central retina of the right eye was caused by the knockout of *CNGB3*, rather than the puncture. These findings demonstrated the disease phenotype of cone dysfunction in the NHP model.

### Evaluation of the SpCas9 Residue in Other Tissues

To test long-term residue of AAV-SpCas9 in tissues other than retina, one NHP was maintained until 1 year after injection. We examined the tissue residue of SpCas9 in both the DNA and RNA levels by PCR and reverse transcription PCR, respectively. A px330-vector containing SpCas9 open reading frame was used as the positive control. As a result, the CRISPR-Cas9 was not detectable in the heart, liver, spleen, kidney, brain, testis, or blood (Figure S6). These results demonstrated that the subretinally delivered AAV9s remained exclusively in the retina and could not traverse the blood-retina barrier, implying the safety of the AAV-mediated CRISPR-Cas9 system.

## Discussion

The *CNGA3* and *CNGB3* genes encode the α and β subunits of the cone cyclic guanosine monophosphate (cGMP)-gated cation channel in cone photoreceptors.^27^ The *GNAT2* gene encodes a protein subunit involved in cone transduction.^9^ Kohl et al.^28^ reported that the 1148delC mutation accounted for over 70% of all *CNGB3* mutant alleles in patients with achromatopsia. Similarly, Ding et al.^26^ generated *CNGB3* ^−/−^ mice with cone dysfunction at postnatal day 30; further, Sidjanin et al.^29^ reported canine cone degeneration from *CNGB3* mutations, which was orthologous to the human achromatopsia locus ACHM3. In this study, we generated a somatic *CNGB3* knockout model in NHPs to recapitulate achromatopsia through subretinal injection of AAV-mediated CRISPR-Cas9 targeting *CNGB3*. The indel ratio detected in deep sequencing was low, and we speculate there are two possible explanations: (1) the component of the AAVs in the area of retina dissected for deep sequencing was poorly enriched in the central macula, or (2) in contrast to immunohistochemistry and single-cell sequencing in DsRed^+^ cones, deep sequencing was performed with a large number of cells, including uninfected cells. Even so, we detected a considerable decrease of *CNGB3* in the cones. A targeting efficiency of 12%–14% was suggested by both immunohistochemistry and by transcription-level analysis, and a consistently reduced mfERG response was also observed, suggesting cone dysfunction in the central macula.

There are several gene therapies reported in achromatopsia animal models: sheep, and mouse models with *CNGA3-*related achromatopsia,^30^^,^^31^ mouse and canine models with *CNGB3* mutations,^32^, ^33^, ^34^ and a mouse model with a genetic defect in the *GNAT2* gene.^35^ However, all of those models were based on macula-free animals with retinas that are physiologically different from those of humans. In contrast, in their genetic and phenotypic characteristics, NHPs are the species most similar to humans.^18^ Primate models for numerous diseases are in great demand, but they have been either unfeasible in the past because of technological bottlenecks, policy restrictions, or high cost.^18^^,^^36^ Importantly, NHPs exhibit a very low reproduction rate, which has presented a significant challenge. Humans and most NHPs exhibit a typical retinal structure that is composed of high-density cones referred to as the macula.^17^ However, this structure does not exist in the most commonly used animal models, including rodents, felines, and canines. There were two studies exploring cerebral achromatopsia in NHPs by surgical ablation of a cortical region.^37^^,^^38^ Apparently, they were not NHP models with the same heritable achromatopsia as that found in human patients. The lack of suitable animal models has hindered the progress of the study of macular-related disease mechanisms.

Currently, we can obtain inherited retinal-degeneration NHP models either by seeking spontaneous-mutation models or by generating genetically engineered models. Whole-genome sequencing in 21 rhesus macaques discovered 47 variants that are identical to variants previously implicated as pathogenic in humans, indicating the potential of NHPs being the novel human disease models.^39^ Francis et al.^40^ genotyped a cohort of 137 unrelated rhesus macaques with and without macular drusen and revealed that rhesus monkeys share common susceptibility genes for age-related macular disease with humans. Ikeda et al.^41^ performed large-scale screening of a cohort of 1,443 cynomolgus monkeys and discovered among the pedigreed population two individuals in a cynomolgus monkey family with retinitis pigmentosa. Through whole-exome sequencing, a single-nucleotide polymorphism (SNV) in *CCDC13* and an indel in *CEP164* were reported to possibly be responsible for the disease. Moshiri et al.^42^ identified four related rhesus macaques with visual impairment and finally confirmed a homozygous R565Q missense mutation in the catalytic domain of PDE6C, a cone-specific phototransduction enzyme associated with achromatopsia in humans. This is a typical case of a nonsyndromic NHP model of retinal degeneration. Coincidentally, Peterson et al.^43^ reported cases of Bardet-Biedl syndrome in rhesus macaques due to a mutation in *BBS7.* However, they only identified five cases. We also screened a cohort of hundreds of rhesus macaques, and six individuals were diagnosed with oculocutaneous albinism without fovea.^44^ We further identified a biallelic p.L312I mutation in *TYR* and a homozygous p.S788L mutation in *OCA2.*^44^ The spontaneous, inherited retinal degeneration of NHP models is described in the literature, but they are too rare to meet research needs. Hence, genetically engineered monkey models are especially sought after. The first transgenic monkey was created by retroviral gene transfer into mature oocytes, but that method exhibited a low efficiency.^45^ Thereafter, transgenic NHP models were introduced into the study of human diseases.^46^, ^47^, ^48^ Those transgenic NHP models were controversial because their endogenous gene copies were still functional. We focused on *CNGB3* in loss-of-function NHP models to mimic macular disease in human patients. In the past decade, the CRISPR-Cas9 gene-editing system has rapidly dominated the field of genetic engineering.^19^^,^^20^^,^^22^^,^^49^, ^50^, ^51^, ^52^, ^53^ Before the development of this technology, the genetic engineering of primates was a laborious process with an extremely limited editing efficiency.^54^^,^^55^ Researchers have succeeded in generating NHP models of neurological diseases such as Rett syndrome, autism, and Parkinson’s disease.^46^^,^^47^^,^^56^ Unlike the brain, the macula has a diameter of approximately 5.5 mm, and it is a very small, specialized tissue in primates.^17^ This trait of the macula allowed us to generate NHPs with macula-specific gene knockout through the injection of AAV-mediated CRISPR-Cas9 vectors. Notably, NHP macula lutea exhibited a retinal structure that was extremely similar to that of humans. Therefore, NHPs are better models for monitoring retinal disease progression than other animals. Herein, we developed a new NHP model using AAV-CRISPR-Cas9-mediated ablation of *CNGB3* in a proportion of macular cones.

In this study, we used 4-year-old cynomolgus macaques, which are sexually mature, and their physiological statue tended to be stable. Macaques in this period are equivalent to teenagers aged at 12–16 years in human, which is when many macular degenerative diseases have their onset. Of course, we could re-arrange the injection window to match the “critical period” during which the disease develops. The methodology implicated has succeeded in generating partial *CNGB3* knockout in NHP models. Although some spot damages remained, they did not ruin the integrity of the fovea, which is responsible for central vision. The target efficiency reached to 12%–14%, leading to a significant reduction in mfERG response, consistent with achromatopsia in humans,^57^ indicating cone dysfunction in the NHP models.

The AAV9 used in this study achieved comparable infection, and the spCas9 system produced a 12%–14% gene-editing rate. We used spCas9, rather than *Staphylococcus aureus* Cas9 (saCas9), because there were more spCas9 protospacer adjacent motifs (PAMs) in exon 6. Because the capacity of an AAV vector is limited, the spCas9s have to be driven by elongation factor (EF) promoter, which is small but has relatively low efficient. A possible future strategy to increase the gene-editing rate is to combine multiple sgRNAs to target more sites during one injection or to increase the injected dose. A shortcoming of our method of generating a macular degeneration model is that the mutations in the NHP cone photoreceptors are inconsistent because non-homologous end joining (NHEJ) occurs randomly in each cell. Undesirable off-target effects might affect the identities of AAV-infected cones as well. Alternatively, NHP models produced through other methods, such as the targeting of genes in the germline and the screening of spontaneous mutations in monkey pedigrees, are free of such concerns. Those two methods give rise to uniform mutations in each cell, which can be used in both cell-based and gene-based treatment studies. However, the time-consuming nature of those approaches is their most obvious and is the greatest challenge in relation to somatic-based model generation. Nevertheless, somatic-based methods cause mutations and cell death and eventually lead to macular degeneration. For cell replacement analysis, this inconsistency has no influence on application prospects because these cells can be easily distinguished from host cells through the construction of a color-based reporter system in donor cells. Moreover, the cell death ratio and the mutation rate of the cone photoreceptor are controllable through the injection of different doses.^58^ In addition, the AAV-vector-infected areas are region restricted, allowing us to generate an NHP somatic knockout model.

Our study also provides important evidence for the tolerance of CRISPR-Cas9-based *in vivo* gene therapy targeting the retina in primates. Through long-term follow-up of the sham-vector-injected eyes at post-injection day 90, we found that NHP retinas could tolerate the AAV treatment without any structural or functional damage. The targeting efficiency observed in the targeted eye is notable, reflecting promising prospects for future pre-clinical use. Although several clinical studies of retinal diseases have been initiated,^59^, ^60^, ^61^ sufficient evidence from preclinical studies on NHPs is strongly recommended. Tobias et al.^62^ and Jacobson et al.^63^ demonstrated the safety of AAV-based subretinal administration, and our results support the efficiency of AAV9-CRISPR-Cas9-based gene correction in retinal cells,^64^^,^^65^ encouraging us to perform precise gene editing *in vivo*. With improvement in gene-editing efficacy and accuracy, CRISPR-Cas9 can be used as a scalpel in the treatment of macular diseases.

In conclusion, we, for the first time, generated an NHP model with a partial knockout of the *CNGB3* gene in the macular cones, exhibiting cone dysfunction consistent with achromatopsia in human patients. This new strategy of gene knockout in macular cones of NHPs provides a rapid way to establish retinal disease models in NHPs.

## Materials and Methods

### Animals

Three healthy cynomolgus monkeys that were 4 years old were used in this study. All animals were housed at an Association for Assessment and Accreditation of Laboratory Animal Care (AAALAC) accredited facility. The animal protocols used were approved in advance by the Institutional Animal Care and Use Committee. Monkey 1 and monkey2 were sacrificed at day 150 for single-cell sequencing and immunofluorescence. For long-term observation, monkey 3 was raised until to 1 year after injection.

### Plasmid Constructs

The sgRNAs were designed with the Cas-Designer platform (http://www.rgenome.net/cas-designer/). Oligonucleotides were synthesized, phosphorylated, annealed, and ligated into the BbsI sites of the px330 plasmid (Addgene: 42230). SpCas9 with a FLAG-epitope tag driven by the EF promoter was subcloned into the AAV plasmid (Figure S7A) provided by IDMO (IDMO, Beijing, China). China National GeneBank (CNGB)-sgRNA3, driven by the U6 promoter, was cloned into an AAV vector (Figure S7B) containing cytomegalovirus (CMV)-DsRed expression cassettes provided by IDMO. The AAVs were packaged and purified by IDMO.

### Cell Culture and Transfection

COS-7 cells (provided by IDMO) were maintained in DMEM (GIBCO, Carlsbad, CA, USA) supplemented with 10% heat-inactivated fetal bovine serum (FBS) (GIBCO, Carlsbad, CA, USA) and 100 μg/mL penicillin/streptomycin with incubation at 37°C under 5% CO_2_.

COS-7 cells were seeded into six-well plates and transfected with FuGENE HD reagent (Roche, Basel, Switzerland) following the manufacturer’s instructions. A total of 2 μg of plasmids was added to each well of the six-well plate.

### T7E1 Assay

Genomic DNA was extracted with a Blood/Cultured Cell DNA Kit (Simgen Biotech, Hangzhou, China) following the manufacturer’s recommended protocol. The regions surrounding the CRISPR target site were amplified with Phanta Max Super-Fidelity DNA Polymerase.

The PCR product was denatured at 95°C for 5 min and gradually reannealed to form DNA heteroduplexes. The heteroduplexes were digested with T7 endonuclease I (NEB, Shanghai, China) following the manufacturer’s recommended protocol. Cleaved DNA fragments were separated in agarose gels, and the intensity of each band was measured with ImageJ software. The percentage values of the indels were calculated as described. The expected size of the unedited PCR product was 700 bp, and the edited product was 300 bp and 400 bp.

### FP and FFA

Anaesthesia was performed with 50 mg/mL of ketamine and 0.5 mg/mL of atropine, and the pupils were dilated with Mydrin eye drops. The monkey fundus and FFA were studied with a retinal camera (rtx1, Canon, Tokyo, Japan). The eye position was aligned to the camera by the operator, and color-fundus photos were obtained with the macula located in the center field. After the fundus images were obtained, the monkeys wore hard contact lenses, and artificial teardrops were administered. The monkeys received an intravenous injection of 0.5% fluorescein sodium solution (Alcon, Geneva, Switzerland) at a dose of 20 mg/kg. FFA images of the monkeys were captured for more than 5 minutes.

### OCT

OCT images were studied with Spectralis OCT (Heidelberg Engineering, Heidelberg, Germany). The monkeys were anaesthetized, and their eyes were dilated. Additionally, hard contact lenses were worn to keep the corneas moist. The retinal structure at the injection points was recorded in a 19-line sequence. After the test, the monkeys covered with an electric heating blanket until they revived.

### ffERG and mfERG

ffERG responses in both eyes of three monkeys were recorded at four time points from post-injection day 30 to 120. The monkeys subjected to the ERG experiment required 20 minutes of dark adaption before the analysis. Their body temperatures were maintained at approximately 37°C with an electric blanket. Their pupils were dilated with 1% atropine eye drops and 2.5% phenylephrine hydrochloride eye drops, and the cornea was kept moist by the addition of several drops of methylcellulose solution. Ag/AgCl wire loop electrodes were placed on the cornea of each eye of the monkeys. Standard scotopic and photopic ERGs were recorded using an Espion stimulator (Diagnosys, Lowell, MA, USA). The stimulation was performed according to the International Society for Clinical Electrophysiology of Vision (ISCEV) guidelines. The 0.01, 3.0, and 10.0 scotopic ERG; 3.0 photopic ERG; and 3.0 flicker ERG were recorded.

A multifocal electroretinogram was performed with a D2/5 diagnosis system. The pupils were dilated with 1% atropine eye drops and 2.5% phenylephrine hydrochloride eye drops. The following anesthesia was administered: 50 mg/mL of ketamine and 0.5 mg/mL of atropine. The body position was adjusted, so that the macula fit to the cathode ray tube (CRT) simulator. Data from the right and left eyes were recorded. The mfERG responses were obtained with the supporting software and a scaled array of 61 hexagons. The stimulus luminance was 1,000 cd/m^2^. The pass cutoff was 10–100 Hz. The P1 density (nV/deg^2^) was the main data point examined.

### Isolation of Single-Cone Photoreceptors

The eyes were dissected from the monkeys after they were euthanized and were then transferred to 1X Hank’s balanced salt solution (HBSS) without Ca^2+^ and Mg^2+^. The cornea and lens were removed, and the retina was carefully dissected. DsRed^+^ regions of the retina were identified using florescence microscopy (Life Technologies, USA). Small pieces of the DsRed^+^ signal retinas from the right eyes of two monkeys (Figure S2, shown as red circle) were digested with heat-activated papain (Worthington Biochemical, Lakewood, NJ, USA) for 10 min. Digestion was terminated by adding 2% FBS medium, diluted with HBSS, and the retinal tissue was then washed twice with 2% FBS. The digested retina was dispersed into a cell suspension using a 1-mL RNase-free pipette, in which bubbles were avoided. The cell suspension was filtered with a 35-μm nylon mesh (BD Falcon, BD, Franklin Lakes, NJ, USA), followed by centrifugation at 3,000 × *g* for 2 min. The cells were resuspended in 1 mL of 2% FBS and further diluted 20 times with 2% FBS medium. The single-cell suspension was transferred to a 35-cm cell culture dish and screened under a fluorescence microscope. For these single cones, only those that were DsRed^+^ and retained nearly intact structures, including the inner segment, outer segment, axon, and axon terminal, were selected. In addition, cones with intact structure but no positive DsRed signal were captured as controls. The targeted single-cone photoreceptors were captured using a TransferMan 4r micromanipulator (Eppendorf, Hamburg, Germany) with a glass pipette. To prevent negative effects of the cell suspension medium, the cells were first washed using a 1X phosphate-buffered solution (PBS) and then one to three cells were transferred into 2 μL lysis buffer stored in a 200-μL centrifuge tube. The lysis buffer including single cells was kept at −80°C until single-cell sequencing.

### Targeted Deep DNA Sequencing

DNA was extracted from the remaining retina after isolation of the single cells. Fragments of PCR amplified from the on-target site (almost 300 bp) were analyzed by deep sequencing. The PCR products were gel purified using a Monarch DNA Gel Extraction kit (NEB) and were quantified with a Qubit 3.0 Fluorometer (Life Technologies, CA, USA). DNA libraries were constructed with a TruSeq DNA Library Prep kit following the manufacturer’s DNA sample preparation guide. Paired-end sequencing of the DNA libraries was performed on an Illumina HiSeq X platform.

### Immunohistochemistry

The injected retinal areas of the monkeys were checked by fluorescence microscopy, and the central part containing the fovea (Figure S2, shown as yellow ellipse) was dissected and fixed in 4% (wt/vol) paraformaldehyde in PBS for 2 h. Retina pieces were then dissected and re-fixed in 4% (wt/vol) paraformaldehyde for an additional 20 min. After washing in PBS, the retinas were place in 30% (wt/vol) sucrose and embedded in an embedding medium (Neg-50, Thermo Fisher Scientific). Slides containing 30-μm-thick cryosections were blocked in blocking buffer composed of 4% (wt/vol) bovine serum albumin (BSA) and 0.5% (vol/vol) Triton X-100 for 2 h in PBS. Primary antibody treatment of the cryosection slides was performed at 4°C overnight, and incubation with the corresponding secondary antibody was carried out at room temperature for 1 h. The primary and secondary antibodies used were as follows: rabbit anti-M-opsin (1:500 Millipore), rabbit anti-S-opsin (1:500 Millipore), goat anti-CNGB3 (1: 200 Santa Cruz), donkey anti-rabbit immunoglobulin G (IgG) conjugated with Alexa Fluor 594 (1:200; Jackson ImmunoResearch Laboratories), and donkey anti-goat IgG conjugated with Alexa Fluor 488 (1:200; Life Technologies.). The samples were stained with 4,6-diamidino-2-phenylindole (DAPI) and visualized with a laser-scanning confocal microscope (LSM710 Zeiss, Oberkochen, Germany).

### RNA Sequencing of Single-Cone Photoreceptors

To observe transcriptome changes within single cones, the Smart-Seq2 technique, which is a full-length RNA-seq method, was used. The detailed process was described previously.^66^ Briefly, 1 μL of the oligo-dT30VN primer (10 μM) and 1 μL of deoxyribonucleotide triphosphate (dNTP) mix were added to the cell lysis buffer, and the sample was then incubated at 72°C for 3 min to ensure complete mRNA release.

For reverse transcription, 5.7 μL of first-strand cDNA mix (100 U SuperScript II reverse transcriptase, 1 μM template switch oligo (TSO) primer, 5 mM DTT, 1 M betaine, 6 mM MgCl, and SuperScript II buffer) was added to the above sample. The samples were incubated at 42°C for 90 min, followed by 10 cycles of 2 min at 50°C and 42°C, and the enzyme was finally inactivated at 70°C for 15 min. For the amplification of cDNA, the KAPA HotStart PCR kit (KAPA Biosystems) and the IS primer (5′-AAGCAGTGGTATCAACGCAGAGT-3′) were used. The procedure began with denaturation at 98°C for 3 min, followed by 18 amplification cycles with annealing at 67°C for 15 s, and finally ended with extension at 72°C for 5 min.

The amplified cDNA was purified with Agencourt Ampure XP beads (Beckman Culter, USA). The quality of the cDNA library was evaluated with an 2100 Bioanalyzer (Agilent Technologies, USA). Tagmentation of the sample was performed with an Illumina Nextera XT DNA sample preparation kit (Illumina, USA). The amplification of adaptor-ligated fragments was performed with the Nextera XT kit. The libraries of different samples were pooled, and 150-bp paired-end sequencing was then performed on the Illumina HiSeq 2500 platform.

The reads from single-cell sequencing were aligned to the reference cynomolgus monkey genome (Macaca_fascicularis_5.0) using Hisat2 software. Gene expression matrices were generated with a StringTie assembler.

### Statistical Analysis

All statistical data were analyzed with GraphPad Prism 5 and are presented as the means ± standard error of the mean (SEM). p values were calculated using paired and unpaired two-tailed t tests. A value of p <0.05 was considered significant. The labels in the figures are as follows: ∗p < 0.05, ∗∗p < 0.01, ∗∗∗p < 0.001, and n.s, no significance.

### Data Availability

The data that support the findings of this study are available from the corresponding author upon reasonable request.

## Author Contributions

Z.-B.J. and Q. Liu designed the project; J.-N.L., Q. Lin, K.-C.W. performed the experiments; Q. Lin and K.-C.W. performed the data analysis; Z.-B.J., Q. Lin, J.-N.L., and C.-J.Z. wrote the manuscript; and Z.-B.J. and Q. Liu revised the manuscript.

## Conflicts of Interest

The authors declare no conflict of interests.

## Supplemental Information

Document S1. Figures S1–S7 and Tables S1 and S2

Document S2. Article plus Supplemental Information
