# Supplementary material for: Generation of Nonhuman Primate Model of Cone Dysfunction through In Situ AAV-Mediated CNGB3 Ablation
Source: Mol Ther Methods Clin Dev. 2020 Aug 8;18:869–79. doi: 10.1016/j.omtm.2020.08.007 (PMC7479327; doi:10.1016/j.omtm.2020.08.007)
Supplement: Document S1. Figures S1–S7 and Tables S1 and S2 [file mmc1.pdf]

OMTM, Volume *18*

Supplemental Information

Generation of Nonhuman Primate Model of Cone Dysfunction through *In Situ* AAV-Mediated *CNGB3* Ablation

Qiang Lin, Ji-Neng Lv, Kun-Chao Wu, Chang-Jun Zhang, Qin Liu, and Zi-Bing Jin

Supplemental figures

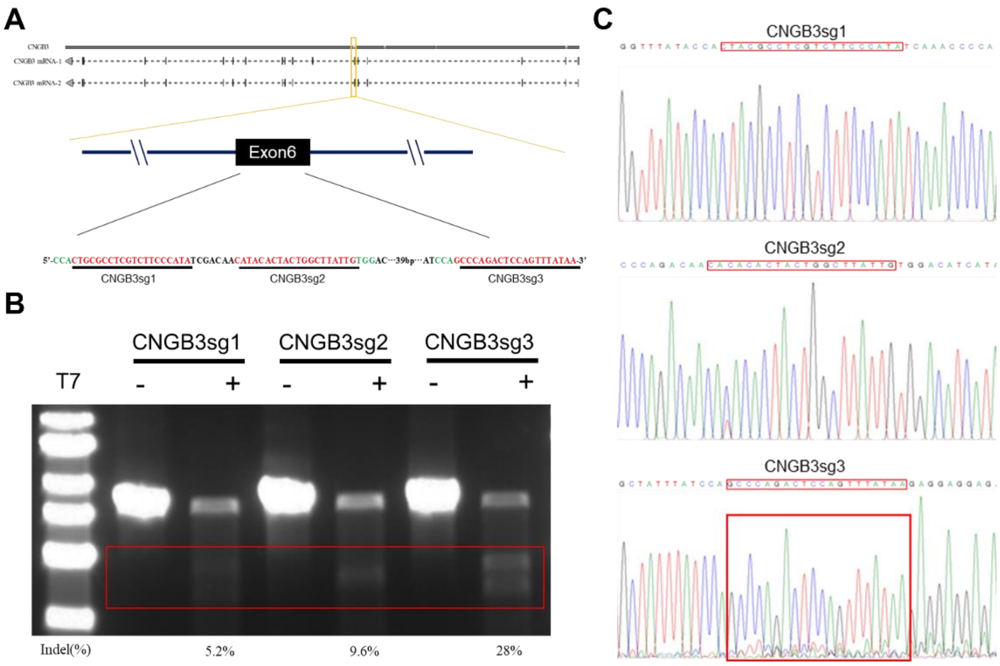

Figure S1. CRISPR-Cas9 targets the *CNGB3* locus in Cos7 cells. (A) Schematic illustration of the *CNGB3* locus and the location of guides employing SpCas9 (red sequence). (B) Representative gel images showing T7E1 assays for Cas9 and sgRNA-mediated indels. Digested production is highlighted in the red box. The ratios of indels are shown at the bottom. (C) Sanger sequencing of target sites of Cos7 cells transfected with CNGB3sg1, CNGB3sg2 or CNGB3sg3. The red box highlights the indels generated by CNGB3sg3.

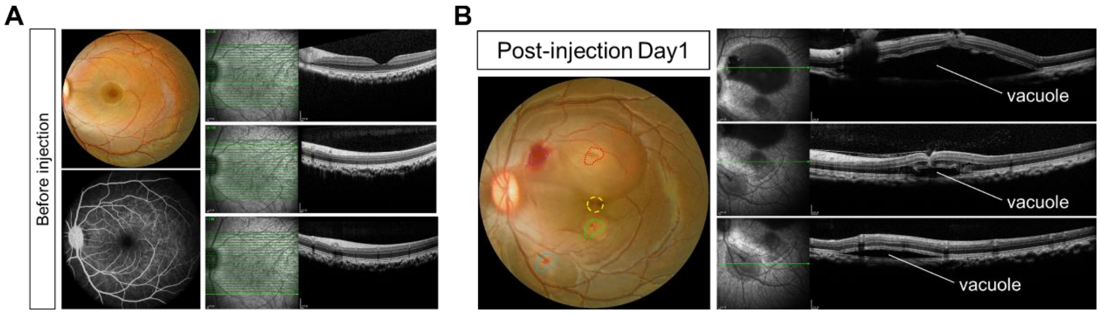

Figure S2. Representative FP, FFA and OCT images of the left eye showing the structure of the retina before and after AAV injection. (A) Representative FP, FFA and OCT images of the left eye before injection. (B) Representative FP and OCT monitoring of the puncture sites and AAV vacuoles on the injection day signed as Day 1. There are four punctures in the left eye that are indicated by colored polygons. The fovea is indicated with a dotted circle in yellow.

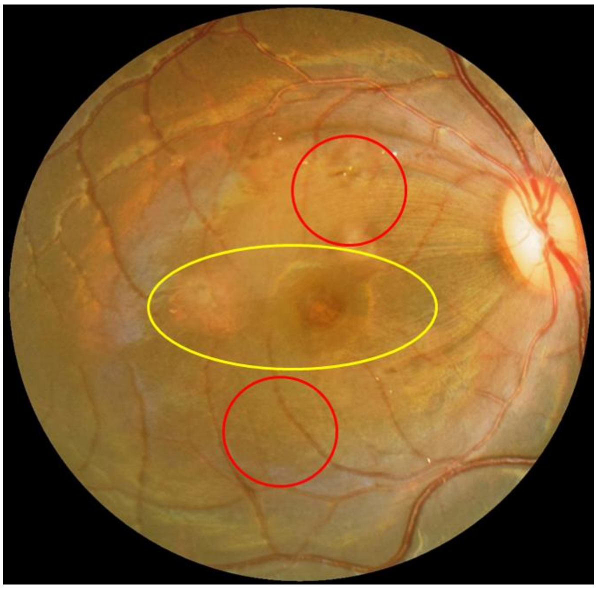

Figure S3. Schematic of retinal dissection. The background shows representative FP images, and the circles indicate the approximate sampling region. The circled part shown in yellow contains the fovea that was used to perform immunofluorescence, and the other two circle parts shown in red were used to isolate single cones to perform single-cell RNA sequencing and deep sequencing.

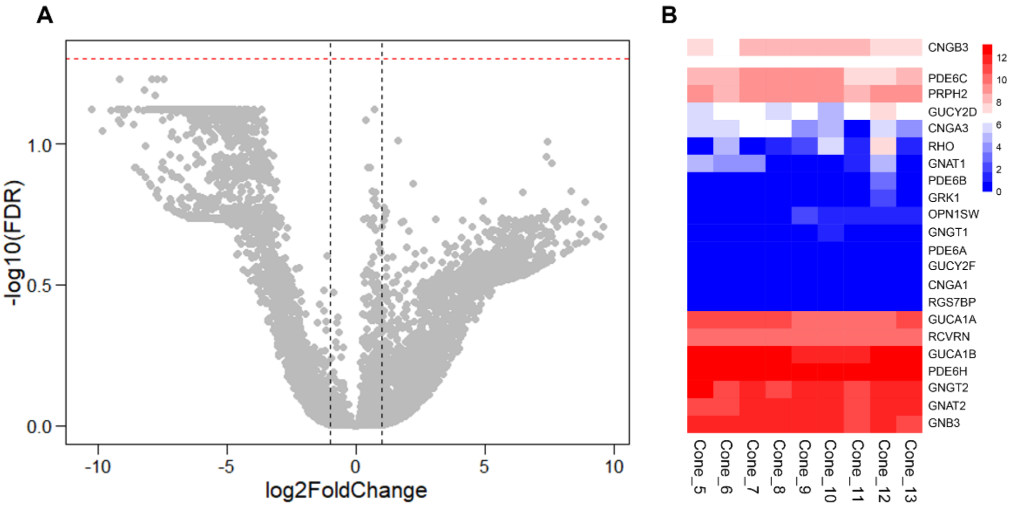

Figure S4. Differentially expressed genes in single-cell sequencing. (A) EdgeR was used to compare sgRNA-positive groups except Cone_6 (8 samples) and control groups (4 samples). As shown in the figure, there were no significant differentially expressed genes. (B) Heatmap of the expression of CNGB3 and its predicted interacting genes. The scale represents $\log_2$(FPKM+1).

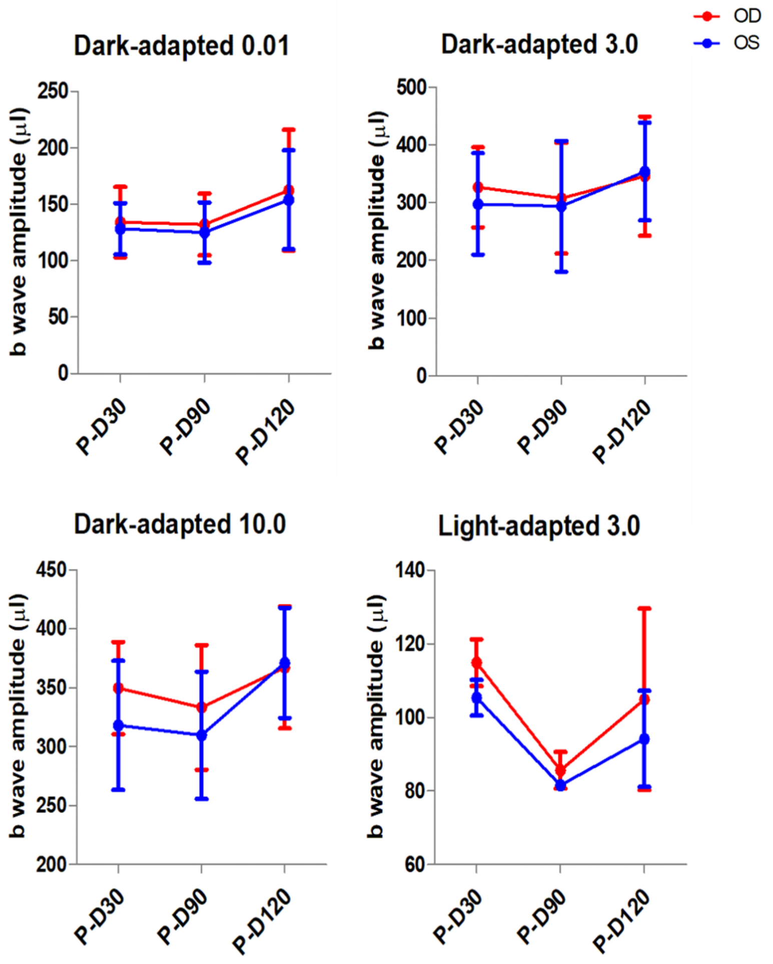

Figure S5. ERG responses of the NHP model after injection. There was no significant difference in dark-adapted and light-adapted ERG responses among NHP models. The P value is calculated by t test.

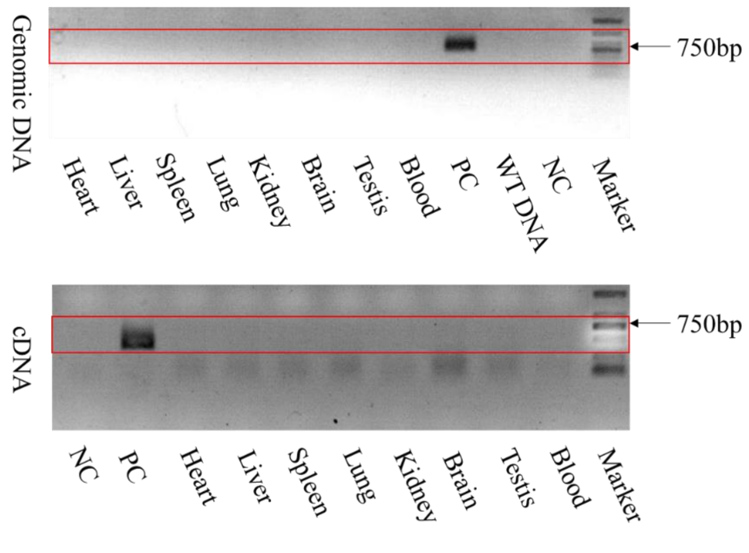

Figure S6. SpCas9 expression in other tissues. The upper image shows the electrophoretic analysis of the PCRs of the Cas9 genome in tissues. The lower panel shows the electrophoretic analysis of reverse transcription PCRs of Cas9 transcripts.

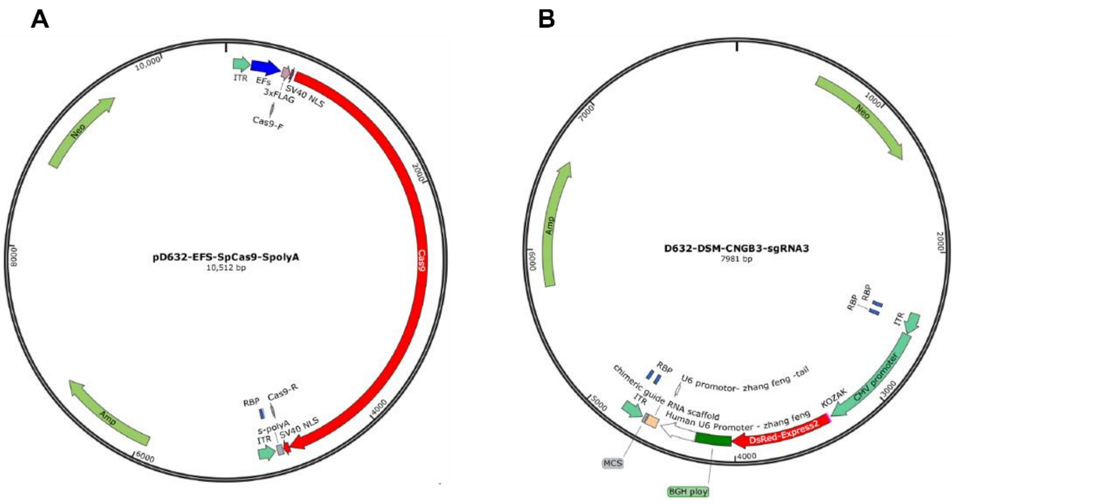

Figure S7. Maps of the AAV9 vector. (A) Map of the AAV-EF-SpCas9 vector. (B) Map of the AAV-CNGB3sgRNA3-DsRed vecto

Supplemental tables

Table S1 Information of single cone groups

	Control group				SgRNA-postive group								
Group	Cone_1	Cone_2	Cone_3	Cone_4	Cone_5	Cone_6	Cone_7	Cone_8	Cone_9	Cone_10	Cone_11	Cone_12	Cone_13
Cell-count	2	3	2	3	1	3	2	3	1	1	1	1	1

Table S2 Genes interact with *CNGB3* predicted in STRING

#node1	node2	node1	node2	node1_external_id	node2_external_id
CNGB3	*GNAT2*	4439069	4434167	9606.ENSP00000316605	9606.ENSP00000251337
CNGB3	*PDE6H*	4439069	4435698	9606.ENSP00000316605	9606.ENSP00000266395
CNGB3	*PDE6A*	4439069	4434424	9606.ENSP00000316605	9606.ENSP00000255266
CNGB3	*GUCY2D*	4439069	4434390	9606.ENSP00000316605	9606.ENSP00000254854
CNGB3	*GNAT1*	4439069	4433558	9606.ENSP00000316605	9606.ENSP00000232461
CNGB3	*GUCY2F*	4439069	4433038	9606.ENSP00000316605	9606.ENSP00000218006
CNGB3	*GNGT1*	4439069	4434061	9606.ENSP00000316605	9606.ENSP00000248572
CNGB3	*GNB3*	4439069	4433453	9606.ENSP00000316605	9606.ENSP00000229264
CNGB3	*RHO*	4439069	4437258	9606.ENSP00000316605	9606.ENSP00000296271
CNGB3	*OPN1SW*	4439069	4434096	9606.ENSP00000316605	9606.ENSP00000249389
CNGB3	*GUCA1B*	4439069	4433500	9606.ENSP00000316605	9606.ENSP00000230361
PDE6C	*CNGB3*	4443750	4439069	9606.ENSP00000360502	9606.ENSP00000316605
CNGA3	*CNGB3*	4446157	4439069	9606.ENSP00000377140	9606.ENSP00000316605
PDE6B	*CNGB3*	4449107	4439069	9606.ENSP00000420295	9606.ENSP00000316605
CNGA1	*CNGB3*	4447097	4439069	9606.ENSP00000384264	9606.ENSP00000316605
GUCA1A	*CNGB3*	4446247	4439069	9606.ENSP00000377784	9606.ENSP00000316605
GNGT2	*CNGB3*	4449343	4439069	9606.ENSP00000426022	9606.ENSP00000316605
CNGB3	*PDE6C*	4328598	4321580	9544.ENSMMUP00000032058	9544.ENSMMUP00000019304
CNGB3	*RGS7BP*	4328598	4326328	9544.ENSMMUP00000032058	9544.ENSMMUP00000027816
CNGB3	*RCVRN*	4328598	4324087	9544.ENSMMUP00000032058	9544.ENSMMUP00000023775
CNGB3	*CNGA1*	4328598	4315094	9544.ENSMMUP00000032058	9544.ENSMMUP00000007610
CNGB3	*GRK1*	4328598	4316303	9544.ENSMMUP00000032058	9544.ENSMMUP00000009743
CNGB3	*GNGT2*	4328598	4312643	9544.ENSMMUP00000032058	9544.ENSMMUP00000003274
CNGB3	*PRPH2*	4328598	4315021	9544.ENSMMUP00000032058	9544.ENSMMUP00000007500
